# Governance of food safety in China's pre-made dishes industry: legal reforms, policy strategies, and international perspectives

**DOI:** 10.3389/fnut.2025.1702278

**Published:** 2025-11-28

**Authors:** Shuchen Tang, YuLong Yang, Zilong Li

**Affiliations:** 1 School of Criminal Investigation, People's Public Security University of China, Beijing, China; 2 School of Law, Beijing Technology and Business University, Beijing, China

**Keywords:** pre-made dishes, food safety, governance, certification, traceability, China, comparative policy

## Abstract

The rapid growth of the pre-made dishes (PMD) industry in China has created new opportunities for rural revitalization and food industry modernization. However, food safety concerns remain a critical challenge, manifested in the absence of a unified standard system, incomplete certification mechanisms, flaws in traceability, and weaknesses in regulatory enforcement. This study conducts an empirical investigation of China's central and local legal documents, complemented by case analysis of recent food safety incidents, to identify the systemic gaps in governance of PMDs. Drawing on macro-, meso-, and micro-level perspectives, we propose a governance framework that integrates compulsory and voluntary standards, multi-stakeholder participation, and digital traceability platforms. Comparative insights from the European Union and the United States further highlight how China's regulatory innovations may contribute to the global discourse on ready-to-eat food safety. The findings suggest that legal and regulatory reform, supported by technology-driven solutions, is essential for ensuring consumer trust and sustainable industry development.

## 1 Introduction

“Food safety is not only a public health issue but also a matter of social trust and governance” (1). Few sectors exemplify this more conspicuously than the rapid expansion of pre-made dishes (PMDs) within China's food system. Once a niche market segment, PMDs have evolved into an indispensable component of daily diets for millions of households, delivering convenience to urban consumers while unlocking new economic opportunities for rural producers. In response to this growth, recent policies such as the 2024 “Notice on Strengthening Food Safety Supervision of Pre-made Dishes and Promoting High-Quality Industrial Development,” jointly issued by the State Administration for Market Regulation (SAMR) and the National Health Commission (NHC), underscore the government's increasing emphasis on oversight and high-quality development in the sector. This raises a fundamental question: How can regulatory regimes safeguard food safety in an industry whose development outpaces the legal frameworks formulated to govern it?

The upsurge in PMD consumption aligns with a broader global trend toward convenience and ultra-processed foods, which now constitute an increasingly large share of dietary intake worldwide (2). In China specifically, the PMD industry has matured into a multibillion-dollar market, propelled by shifting lifestyles, digital platform penetration, and explicit government support under the rural revitalization strategy (3). Consumer survey data indicate that younger generations—particularly digital natives—exhibit strong purchase intentions toward PMDs (4, 5). However, such enthusiasm is mitigated by prevalent concerns regarding product safety, authenticity, and quality, especially amid recurring food safety scandals (6).

Food safety in China transcends technical considerations to embody a deeply rooted social and political concern (1). Past crises, such as the melamine scandal, have left enduring impacts on consumer trust and elevated food safety to the status of a national policy priority (7). In response, China has implemented comprehensive regulatory reforms, including the enactment of the Food Safety Law and its subsequent amendments, which aim to strengthen pre-market authorization, labeling requirements, and oversight mechanisms (8, 9). Nevertheless, PMDs pose distinctive regulatory dilemmas: their structural product diversity complicates standardization efforts, fragmented certification systems undermine enforcement efficacy, and underdeveloped traceability mechanisms hinder consumer empowerment. These challenges are further exacerbated by local governments' dual role as both industry promoters and regulators, creating inherent risks of conflicting incentives.

At the same time, the PMD sector epitomizes the inherent tension between innovation and regulation. On one hand, it offers avenues for sustainable transformation, including reducing household food waste, optimizing supply chain efficiency, and facilitating rural-urban integration (10). On the other hand, inadequate governance risks amplifying food safety hazards, eroding consumer trust, and stalling long-term industry growth. The intersection of consumer behavior research, risk perception analysis, and food law scholarship underscores the necessity of a multidisciplinary approach to address these complexities (11). International experience provides valuable comparative insights: The European Union (EU), under the General Food Law Regulation, has established risk-based regulatory frameworks that prioritize traceability and Hazard Analysis and Critical Control Points (HACCP) as core pillars of food safety governance. Similarly, the United States, through the Food Safety Modernization Act (FSMA), mandates preventive control measures and shifts primary responsibility for food safety onto producers. In contrast, China's evolving regulatory system for PMDs still relies heavily on state-led enforcement and voluntary industry standards (8, 9). Situating China's PMD governance within this global context enables a more nuanced assessment of its inherent strengths and structural vulnerabilities.

This study argues that ensuring PMD safety in China requires an integrated governance framework that bridges gaps across legislation, certification, traceability, and enforcement. By conducting an empirical investigation of Chinese regulatory policies, industry standards, and case evidence, this paper identifies systemic shortcomings and explores pathways for reform. The analysis adopts a macro–meso–micro analytical lens: at the macro-level, it examines overarching governance philosophies; at the meso-level, it analyzes institutional structures; and at the micro-level, it evaluates specific rule design. By contextualizing China's challenges within international debates on ready-to-eat food governance, this article contributes both theoretical insights and practical policy recommendations. In doing so, the paper offers three key contributions: First, it enriches existing literature by focusing on the underexamined PMD sector—a rapidly expanding but under-regulated domain within China's food system. Second, it integrates legal analysis, empirical case studies, and international comparison to provide a multidimensional perspective on PMD governance. Third, it anticipates critical debates on balancing innovation with risk regulation in global food systems. Addressing these issues is pivotal for securing consumer trust, safeguarding public health, and promoting the sustainable development of China's PMD industry.

## 2 Materials and methods

### 2.1 Research design

We employed a qualitative, document-based design combined with structured comparative policy analysis to examine systemic challenges in the governance of PMDs in China. A multi-method strategy is warranted because PMDs sit at the intersection of evolving consumption patterns, food law, and risk perception. Guided by a macro–meso–micro framework, we analyzed: (i) macro-level governance philosophy (e.g., state–market–society co-governance); (ii) meso-level institutional arrangements (e.g., roles of central/local authorities and industry associations); and (iii) micro-level legal rules for standards, certification, traceability, and enforcement (12). To enhance transparency and reproducibility, we followed established procedures for systematic evidence synthesis and qualitative document analysis, including protocol definition, search and screening, coding, and synthesis.

### 2.2 Data sources and sampling

Legal and regulatory corpus. We compiled a corpus of national and provincial regulatory texts, normative guidelines, and enforcement notices pertinent to PMD production, labeling, market access, and supervision (13). Because doctrinal summaries can capture both black-letter requirements and implementation logic, we triangulated primary legal texts with authoritative legal analyses of China's food information and pre-market authorization regimes (see Table 1).

**Table 1:** Data sources and their analytical purpose.

**Category**	**Content**	**Examples**	**Analytical purpose**
Legal and regulatory corpus	National and provincial laws, regulations, guidelines, and enforcement notices	Food Safety Law (2015/2021); Anti-Food Waste Law; provincial PMD measures	Identify statutory requirements, institutional roles, and enforcement logics
Standards and technical norms	Multi-level standards applicable to PMDs (mandatory and voluntary)	13 national, 4 industry, 27 local, 207 group, 12 enterprise standards^*^	Evaluate coherence of standardization system and its alignment with international norms
Cases and governance pilots	Documented PMD incidents, regulatory responses, and local pilot initiatives	The 2024 “3·15” Gala's exposure of the Meicai Kou Rou incident in Anhui exemplifies PMD safety failures, where substandard meat processing violated hygiene standards, prompting regulatory scrutiny; Shandong digital traceability pilot	Illustrate governance gaps, enforcement practices, and innovation in supervision
International comparators	Peer-reviewed syntheses and global frameworks on food safety and traceability	EU General Food Law risk-based regulation; US FSMA preventive controls; blockchain traceability studies	Benchmark China's governance against mature international models

^*^As of January 2024, around 300 standards related to pre-made dishes are currently in force in China, including 13 national standards, 4 industry standards, 27 local standards, 207 group standards, and 12 enterprise standards. The data are drawn from the National Public Service Platform for Standards Information (https://std.samr.gov.cn/gb).

Standards and technical norms. We mapped the multi-level standardization landscape relevant to PMDs—national, industry, provincial/local, group, and enterprise standards—focusing on scope, legal force (mandatory vs. voluntary), and alignment with internationally recognized traceability and safety management concepts (14, 15).

Cases and governance pilots. To examine practice–policy feedback loops, we constructed case vignettes from publicly documented PMD incidents and regulatory responses (e.g., media investigations, administrative actions), alongside provincial pilots for licensing, certification, and digital traceability. We prioritized cases that: (i) involved PMD safety failures or close calls; (ii) triggered discernible regulatory action; and (iii) provided sufficient documentation for process tracing (16). International comparators.

For external benchmarking, we assembled a reference set of peer-reviewed syntheses on food safety management and traceability (EU risk-based approaches, HACCP, supply-chain transparency; US preventive controls), rather than citing statutes without DOIs, to preserve bibliometric traceability and comparability.

### 2.3 Coding and analytical procedures

We adopted a two-stage coding strategy to systematically analyze the regulatory corpus. In the first stage, deductive coding applied the macro–meso–micro framework, categorizing legal and policy provisions by governance level (e.g., macro: co-governance mechanisms; meso: role clarity and institutional mandates; micro: certification, traceability, and enforcement). In the second stage, inductive thematic coding was employed to capture emergent patterns not anticipated in the initial framework, such as the fragmentation of voluntary standards or tensions between industrial promotion and regulatory oversight. Coding was conducted iteratively using a constant comparison approach, enabling refinement of categories and resolution of overlaps as the analysis progressed. To ensure reliability, two researchers independently coded a stratified 20% subsample of documents. Discrepancies were reconciled through discussion, and the codebook was subsequently revised before full-set coding proceeded (17).

For the case analysis, we applied a structured, focused comparison template that covered five dimensions: problem characterization, regulatory instruments invoked, implementation actors, information disclosure and traceability elements, and observed outcomes. This template facilitated systematic cross-case synthesis and enabled the identification of recurrent governance bottlenecks, such as weak deterrence and coordination failures.

Finally, the comparative policy analysis followed a “most similar systems” design, contrasting China's PMD governance arrangements with mature risk-based frameworks documented in the international literature. Four criteria guided the comparison: hazard prevention tools, traceability architecture, allocation of responsibility, and transparency provisions. This approach allowed us to isolate institutional differences that shape regulatory effectiveness while controlling for similarities in broader food safety objectives.

## 3 Results

This section may be divided by subheadings. It should provide a concise and precise description of the experimental results, their interpretation, as well as the experimental conclusions that can be drawn.

### 3.1 Structure of analysis and coding results

To ensure analytical clarity, the results are organized according to the macro–meso–micro framework introduced in Section 2. This layered approach allows for a systematic evaluation of China's governance of PMDs, capturing both the structural design of the legal system and the specific operational rules applied in practice.

At the macro-level, the focus lies on the overarching governance philosophy that underpins China's food safety regulation (18). Since the promulgation of the Food Safety Law of the People's Republic of China in 2009, and its subsequent amendments in 2015 and 2021, China has progressively shifted from a fragmented, department-led regulatory approach toward a unified framework emphasizing risk prevention, whole-chain supervision, and social co-governance. This philosophy is reflected in national strategies linking food safety to public health, consumer trust, and even political legitimacy.

The meso-level analysis concerns institutional structures and regulatory arrangements. China's food safety governance is characterized by a multi-tiered system involving central authorities (e.g., the State Administration for Market Regulation, the National Health Commission), provincial market regulation bureaus, and local governments (19, 20). Industry associations and enterprises also play a role through the formulation of voluntary standards and certification practices. This institutional layering produces both opportunities for localized innovation and risks of inconsistency, particularly in the emerging PMD sector where national-level guidance remains limited.

At the micro-level, the analysis focuses on the design of specific rules and mechanisms, namely:

Standards: We identified 300 PMD-related standards currently in effect, comprising 35 national, 7 industry, 16 local, 232 group, and 10 enterprise standards (data source: National Public Service Platform for Standards Information, https://std.samr.gov.cn/gb). Importantly, there is no mandatory national or local food safety standard specifically titled “PMDs,” meaning that safety assurance currently relies on general food safety standards (e.g., National Standard for Limits of Pathogenic Bacteria in Prepackaged Foods, Local Food Safety Standard for Convenience Dishes, Local Food Safety Standard for Prepackaged Refrigerated Meals) and recommended PMD-specific standards such as the Code of Practice for Cold Chain Distribution of Pre-made Dishes and the Code of Conduct for Production and Processing of Pre-made Dishes (21).

Certification: Coding of regulatory texts showed that certification requirements are confined mainly to production licensing. No compulsory HACCP-based certification or preventive control systems are in place for PMDs, leaving compliance dependent on voluntary adoption of international standards (e.g., ISO 22000) by larger enterprises.

Traceability: Article 42 of the Food Safety Law mandates traceability, yet our coding revealed that provisions specific to PMDs remain absent. Provincial pilots (e.g., Shandong's QR code-based digital traceability) were identified, but they lack harmonization and national integration.

Regulation and enforcement: Textual analysis of enforcement notices indicates uneven regulatory activity across provinces (22). Shandong and Zhejiang issued PMD-specific guidelines, while most other provinces rely solely on general food safety provisions. Coding also highlighted weak sanctioning clauses, with penalties often limited to administrative fines insufficient to deter repeated violations.

The coding exercise covered 82 legal and regulatory documents (including national laws, provincial measures, and key standards), which were systematically tagged according to the macro–meso–micro framework (see Table 2). Results revealed that 42% of provisions related to general food safety philosophy and governance principles (macro-level), 33% addressed institutional allocation of responsibilities and regulatory procedures (meso-level), and only 25% directly concerned concrete PMD-specific mechanisms such as standards, certification, and traceability (micro-level). This distribution highlights a disproportionate emphasis on overarching governance rhetoric, with insufficient legal detailing for emerging food categories like PMDs (23).

**Table 2:** Coding distribution of regulatory and standard documents related to PMDs.

**Analytical level**	**Definition**	**Number of coded items**	**Proportion (%)**
Macro-level	Governance philosophy, overall principles, and legal frameworks.	34	42%
Meso-level	Institutional allocation of authority, procedures, and regulatory arrangements.	27	33%
Micro-level	Specific operational mechanisms: standards, certification, traceability, enforcement.	21	25%
Total		82	100%

Overall, the results demonstrate that while China's food safety legal system is comprehensive in structure and ambitious in scope, its adaptation to the PMD sector remains underdeveloped. The absence of binding PMD-specific standards, weak certification systems, partial traceability mechanisms, and uneven enforcement collectively form the core governance dilemmas confronting the industry.

### 3.2 Specific deficiencies of PMDs in China

#### 3.2.1 Deficiencies in the standard system

A central deficiency in the governance of PMDs in China lies in the absence of a unified, mandatory national food safety standard specifically tailored for this sector. While food safety legislation at the national level provides broad coverage for general food categories, it has yet to establish binding provisions that address the unique features of PMDs, such as their composite ingredient structures, extended cold-chain logistics, and ready-to-heat or ready-to-cook formats (24). Instead, the current framework relies on a fragmented and layered system consisting of national, industry, local, group, and enterprise standards. However, the vast majority of these are voluntary rather than compulsory, meaning that enterprises can selectively comply depending on commercial interests, local administrative pressures, or reputational incentives. The reliance on voluntary standards produces a highly inconsistent regulatory landscape. Producers in one province may face requirements that differ considerably from those in another, and even within the same jurisdiction, the applicability of group or enterprise standards may vary based on the influence of particular industry associations or leading companies. This results in a patchwork of technical specifications, covering aspects such as ingredient quality, processing hygiene, packaging, and cold-chain handling, but without ensuring uniformity or nationwide enforceability. Local governments such as Shandong and Guangdong have attempted to fill this gap by issuing pilot standards that cover “ready-to-cook” and “central kitchen” operations, respectively. While these initiatives are valuable as exploratory efforts, they remain geographically limited and do not establish nationwide benchmarks (25).

Industry associations and larger enterprises have also sought to fill the void by formulating group standards. These often focus on highly specific product categories or processing methods, such as standards for braised pork with preserved vegetables, chilled convenience dishes, or cold-chain delivery protocols. Yet, such group standards lack binding force; they operate more as marketing tools or voluntary commitments than as enforceable obligations. This limited scope not only weakens regulatory oversight but also reduces consumer protection, as there is no guarantee that products bearing a “group standard” label meet consistent, enforceable safety benchmarks. The absence of mandatory national standards undermines both regulatory enforcement and consumer trust. From the regulatory perspective, inspectors lack consistent criteria against which to evaluate compliance, resulting in discretion-heavy enforcement practices that vary across provinces and localities. From the consumer perspective, the lack of uniform labeling, disclosure, and safety benchmarks fuels skepticism toward PMDs, especially in the wake of recurring food safety scandals. Moreover, the gap in standardization creates difficulties for inter-provincial trade, as products certified in one jurisdiction may not be readily accepted in another. This not only hampers industrial integration but also slows down the construction of a national market for PMDs (26, 27). In sum, the standardization deficit represents a structural barrier to both effective governance and sustainable industry development.

#### 3.2.2 Weak certification mechanisms

Certification mechanisms form another critical weakness in the governance of PMDs in China. At present, certification requirements for enterprises primarily involve obtaining a production license under the Food Safety Law. While this licensing system is necessary as a gatekeeping mechanism for market entry, its substantive scope is limited. It requires firms to demonstrate compliance with basic facility and procedural requirements, but does not impose comprehensive obligations across the entire food production chain. For instance, it does not mandate detailed hazard analysis, critical control point identification, or routine monitoring protocols that would address the risks unique to PMDs, such as microbial contamination during cold-chain storage or cross-contamination in centralized kitchens. This structural gap leaves the burden of pursuing more advanced certifications—such as HACCP, ISO 22000, or domestic quality management seals—to the voluntary decisions of enterprises. Larger companies, especially those with significant brand visibility and national distribution networks, may choose to adopt such certifications to enhance market credibility and consumer confidence. However, small- and medium-sized enterprises (SMEs), which dominate the PMD sector in both number and market share, frequently avoid these certifications due to their associated costs and administrative burdens (28). The result is a dual structure: a small segment of firms operating under higher-level certifications and a large majority relying solely on the minimum production license.

This imbalance produces highly uneven safety practices across the sector. Larger enterprises are often able to implement traceability systems, supplier audits, and risk management protocols consistent with international best practices, while SMEs continue to operate with minimal oversight, limited documentation, and cost-driven shortcuts. Case studies have repeatedly shown that SMEs are disproportionately represented in food safety scandals, reflecting their lower investment in certification and monitoring systems. This not only exposes consumers to higher risk but also creates competitive distortions, as firms that avoid certification gain short-term cost advantages over those investing in compliance. Moreover, the voluntary nature of certification in China weakens its deterrent effect. Because certifications are not legally mandatory, regulators cannot sanction firms solely for failing to adopt them. This differs from contexts where certification is embedded into legal frameworks, making it an enforceable duty. In China, certification remains a reputational choice rather than a legal obligation, which reduces the incentive for SMEs to pursue it. The absence of compulsory, product-specific certification mechanisms for PMDs therefore represents a systemic vulnerability: it leaves the majority of producers without robust safety management systems, while regulators lack the authority to mandate compliance beyond the minimal licensing requirement (29).

#### 3.2.3 Gaps in traceability systems

Traceability systems are widely recognized as essential for ensuring food safety in complex supply chains, yet in China's PMD industry they remain underdeveloped. Although the Food Safety Law requires producers to establish traceability mechanisms, the actual implementation is inconsistent and often perfunctory. In practice, many PMD enterprises maintain only basic records such as supplier invoices, delivery notes, or batch codes. While these documents provide minimal compliance with legal requirements, they fall far short of enabling effective tracking of raw material origins, production processes, and distribution channels. The limitations of these rudimentary systems are particularly problematic given the nature of PMDs. Unlike single-ingredient products, PMDs typically involve multiple raw materials sourced from diverse suppliers (30). For example, a ready-to-cook dish might contain meat, vegetables, sauces, and condiments, each subject to different safety risks and storage requirements. Without a robust traceability system, identifying the source of contamination or spoilage in such products becomes nearly impossible. This hinders both regulatory interventions and enterprise-led recalls, increasing the likelihood of widespread consumer exposure in the event of safety incidents.

Technological solutions offer potential improvements but remain limited in scope. Several provinces, most notably Shandong, have piloted digital platforms that integrate QR codes or centralized databases to allow regulators and consumers to access supply-chain information. However, these pilots cover only a small subset of enterprises and rely heavily on self-reported data. Without independent verification or uniform national standards, the reliability of such systems remains questionable. Emerging technologies such as blockchain and Internet of Things (IoT)-based monitoring have been discussed in policy and academic circles, but high implementation costs, limited technical capacity, and fragmented governance structures have prevented their large-scale adoption in the PMD industry. The inadequacy of traceability also undermines consumer confidence. Surveys show that consumers increasingly demand transparency regarding the origin and handling of PMDs, particularly in light of recurring scandals. Yet in most cases, consumers lack access to reliable and easily interpretable product information. This information asymmetry not only limits informed consumer choice but also erodes trust in the broader industry. In regulatory terms, weak traceability also hampers the efficiency of inspections and enforcement. Regulators cannot easily trace violations back to specific supply-chain nodes, which reduces the precision and deterrence of enforcement actions. Thus, the gaps in traceability represent a structural weakness that undermines both preventive governance and responsive interventions (31).

#### 3.2.4 Inconsistencies in regulatory enforcement

The final major deficiency concerns inconsistencies in regulatory enforcement. While the Food Safety Law provides a national legal framework, its implementation is delegated to provincial and local authorities, resulting in substantial variation in enforcement intensity and effectiveness. Some provinces, such as Shandong, have taken proactive steps by issuing PMD-specific guidelines, conducting targeted inspections, and piloting digital monitoring platforms. In contrast, many other provinces continue to apply only general food safety provisions, with little sector-specific adaptation or enforcement emphasis. This unevenness creates regulatory gaps and undermines the consistency of governance nationwide. Enterprises operating in provinces with more active oversight are subject to stricter compliance requirements, while those in less regulated jurisdictions may face minimal scrutiny. This creates perverse incentives for regulatory arbitrage, where firms may choose to locate production in areas with weaker enforcement to reduce compliance costs (32). Such disparities not only weaken the credibility of the regulatory system but also hinder the development of a level playing field within the PMD industry.

Another critical issue is the inadequacy of penalties for violations. Enterprises caught engaging in unsafe practices—such as using substandard raw materials, falsifying traceability records, or neglecting hygiene protocols—are frequently penalized with fines or temporary suspensions. However, these sanctions are often far lower than the profits gained from non-compliance, meaning that violations can remain economically rational for firms. The weak deterrent effect of current penalty regimes reduces their capacity to prevent recidivism.

Transparency also poses challenges. Inspection results and enforcement actions are not consistently disclosed to the public, limiting both consumer awareness and the reputational consequences for violators. This lack of openness further reduces the deterrent effect of enforcement and constrains the role of social supervision. Finally, overlapping responsibilities among regulatory agencies can lead to fragmented enforcement, with duplication in some areas and gaps in others. The combination of uneven enforcement intensity, inadequate penalties, limited transparency, and fragmented institutional arrangements creates an enforcement landscape ill-suited to the complex risks posed by PMDs (33).

### 3.3 International experience

The preceding analysis exposed major weaknesses in China's governance of pre-made dishes, ranging from fragmented standards to uneven enforcement. To move from diagnosing these domestic challenges toward identifying potential solutions, the following section draws on international experience, highlighting institutional practices that may inform regulatory improvements in the Chinese context (see Table 3).

**Table 3:** International governance experience relevant to PMDs.

**Dimension**	**EU framework**	**US framework**	**China (current status)**
Assignment of responsibility	General Food Law [Regulation (EC) No 178/2002]: primary responsibility lies with food business operators; penalties must be “effective, proportionate, dissuasive.”	FSMA: firms hold primary duty to prevent hazards; responsibility formalized in written food safety plans.	Responsibility defined broadly in Food Safety Law, but lacks PMD-specific obligations and deterrent penalties.
Risk-based management	Regulation (EC) No 852/2004: mandatory HACCP across all food sectors.	FSMA Preventive Controls Rule: hazard analysis, preventive controls, oversight by qualified individual.	Production licensing only; no binding HACCP/PCQI-like system for PMDs.
Supply-chain verification	Supplier approval and due diligence required under hygiene package.	FSMA Subpart G: formal supply-chain program with verification (audits, sampling, documentation).	No explicit supplier verification duties in PMD governance; relies on general food safety clauses.
Traceability	Regulation (EC) No 178/2002, Article 18: “one step back, one step forward” traceability obligation.	FSMA §204 Food Traceability Rule: critical tracking events and key data elements for designated foods.	Traceability legally required but poorly implemented; pilots in a few provinces (e.g., Shandong).
Official controls	Regulation (EU) 2017/625: risk-based inspections, audits, sampling; strong sanctioning authority.	FDA authority under FSMA: mandatory recall (§423), suspension of facility registration (§415(b)).	Enforcement delegated to provinces; sanctions often limited to fines or suspensions with low deterrence.
Rapid alert/digital modernization	Rapid Alert System for Food and Feed (RASFF): cross-border notification and coordinated action.	FDA “New Era of Smarter Food Safety”: tech-enabled traceability, data interoperability, outbreak forensics.	No national rapid alert or digital traceability network dedicated to PMDs.

#### 3.3.1 Assignment of responsibility

One of the foundational elements in international food safety governance is the assignment of primary responsibility to food business operators (FBOs). In the European Union (EU), Regulation (EC) No 178/2002—widely known as the General Food Law—establishes a clear principle: FBOs are responsible for ensuring that foods under their control meet the requirements of food law at all stages of production, processing, and distribution. This provision is not merely declaratory but also supported by strong enforcement expectations, as Member States are required to establish penalties that are “effective, proportionate, and dissuasive.” The explicit articulation of responsibility ensures that enterprises cannot evade liability by pointing to deficiencies in regulatory inspection or gaps in oversight. Instead, firms are compelled to internalize the costs of safety management as part of their core operational obligations (34).

The United States adopts a parallel, though differently structured, approach through the FSMA. The FSMA significantly expanded the legal responsibilities of food companies by shifting the regulatory paradigm from reactive responses to foodborne illness toward proactive prevention. Under FSMA, covered facilities must develop written food safety plans that include hazard analyses and risk-based preventive controls. These plans must be overseen by a Preventive Controls Qualified Individual (PCQI), ensuring that responsibility is embedded in both institutional structures and individual accountability. By mandating documentation and internal oversight, FSMA closes the loophole where firms might otherwise rely solely on government inspections to validate safety (35).

The assignment of primary responsibility has important implications for sectors like PMDs, which involve multiple ingredients, extended cold-chain logistics, and complex preparation processes. In such settings, relying exclusively on government regulators to detect risks is impractical and inefficient. International experience demonstrates that responsibility must be placed squarely on enterprises, supported by enforceable obligations and credible penalties. In the EU, penalties for violations may include product recalls, business suspension, and financial sanctions sufficient to outweigh any potential economic gains from non-compliance. In the US, the FDA has the authority to suspend a facility's registration, effectively halting its operations. These strong enforcement backstops complement the principle of primary responsibility, making it not only a normative expectation but also a legally enforceable duty.

For China's PMD sector, where responsibility is articulated in general terms under the Food Safety Law but lacks PMD-specific elaboration, international experience suggests the value of clarifying responsibility at the product level. This could involve codifying explicit duties for ingredient vetting, temperature control, allergen management, and labeling, accompanied by penalties proportionate to the risks of non-compliance (36). By learning from EU and US practices, China could move beyond declaratory statements of responsibility and embed enforceable accountability mechanisms into the regulatory framework governing PMDs.

#### 3.3.2 Risk-based management

A second pillar of international food safety governance is the institutionalization of risk-based management systems. In the EU, Regulation (EC) No 852/2004 requires all food businesses to implement and maintain procedures based on HACCP principles. HACCP operates as a science-based, preventive system designed to identify, evaluate, and control hazards significant to food safety. The mandatory nature of HACCP in the EU ensures that risk management is embedded in daily operations, rather than treated as an optional or reputational measure. Moreover, the requirement applies across all food sectors, creating a uniform baseline for safety practices.

In the United States, FSMA similarly requires the adoption of preventive controls grounded in hazard analysis. Facilities must identify and evaluate known or reasonably foreseeable hazards and implement risk-based preventive controls to provide assurances that such hazards will be significantly minimized or prevented. These controls cover a broad spectrum of potential risks, including biological hazards (e.g., Salmonella, Listeria), chemical hazards (e.g., allergens, toxins), and physical hazards (e.g., foreign objects). Importantly, the rule mandates not only hazard identification but also monitoring, verification, and corrective actions, thereby creating a closed-loop system for continuous improvement (37). The requirement that preventive controls be designed and overseen by a PCQI further embeds accountability and expertise into the governance framework.

For PMDs, risk-based management is particularly critical because the products often involve multiple stages of preparation and storage that create diverse hazard points. For example, microbial growth during prolonged cold-chain storage, cross-contamination in central kitchens, or inadequate reheating instructions for consumers can all compromise safety. International experience shows that only a structured, risk-based system like HACCP or FSMA's preventive controls can adequately address such complexities. By contrast, licensing-only systems that focus on facility approval at a single point in time cannot capture the dynamic risks inherent in ongoing operations.

The lesson from EU and US practice is that risk-based management must be legally mandated, uniformly applied, and subject to regular audit and verification. Such systems not only reduce the incidence of foodborne illness but also enhance consumer confidence and facilitate international trade by aligning with globally recognized safety standards. For China's PMD sector, incorporating mandatory HACCP-based procedures or preventive controls would represent a significant step forward in aligning governance with the risk profiles of these products (38).

#### 3.3.3 Supply-chain verification

International regimes also emphasize supply-chain verification as a crucial element of food safety governance. In the US, FSMA requires facilities that rely on their suppliers to control hazards to establish a formal supply-chain program. This includes maintaining an approved supplier list, conducting verification activities such as sampling, records review, or onsite audits, and documenting the entire process. These obligations are codified in 21 CFR Part 117, Subpart G. The aim is to ensure that safety risks are addressed at the earliest point in the supply chain, rather than being left to downstream processors or distributors.

The EU, through its hygiene package, also imposes requirements on operators to exercise due diligence in supplier management. Although structured differently from the US model, the effect is similar: responsibility for ensuring the safety of inputs lies with the enterprise that introduces them into the production process. This approach recognizes that many food safety risks originate upstream, in raw materials such as meat, seafood, or vegetables, which may carry pathogens or chemical residues.

For PMDs, supply-chain verification is particularly salient (39). Given the sector's reliance on multiple raw ingredients sourced from different suppliers, weaknesses at any node in the chain can compromise the entire product. For example, contaminated meat from a small slaughterhouse, pesticide residues in vegetables, or adulterated spices can all propagate through the processing chain into finished PMDs. Without systematic supplier verification, producers cannot guarantee the integrity of their inputs, and regulators may struggle to pinpoint liability when safety incidents occur.

International experience demonstrates that supply-chain verification should not be left to voluntary practice but codified as a legal requirement. This ensures consistency across enterprises and provides regulators with clear benchmarks for inspection. For China's PMD sector, where supplier verification duties are not explicitly mandated, the adoption of such practices would close a critical gap in governance and align the sector with global best practices (40).

#### 3.3.4 Traceability systems

Traceability is another cornerstone of international food safety governance. In the EU, Regulation (EC) No. 178/2002 requires food business operators to implement traceability systems to track food, feed, and raw materials “one step forward and one step backward” at all stages of production, processing, and distribution. Regulation (EC) No. 852/2004 further requires food enterprises to establish an HACCP system and record key information such as raw material usage and the production process. In addition, Regulation (EC) No. 834/2007 sets out relevant requirements for the traceability and labeling of organic products, ensuring specialized oversight for high-risk categories. These layered regulations have proven effective; for instance, the EU's Rapid Alert System for Food and Feed (RASFF) handled 4,301 notifications in 2023, with 58% related to traceability-enabled recalls, enabling swift market withdrawals and reducing outbreak impacts by an estimated 20%−30% compared to pre-2002 levels (41). This obligation ensures that in the event of a food safety incident, the product can be quickly traced and withdrawn from the market. The system is supported by record-keeping requirements and achieves coordination and unification among member states, providing a unified foundation for regulatory authorities and enterprises.

In the US, the FSMA introduced more detailed traceability obligations through the Food Traceability Rule (FSMA Section 204). This rule requires firms handling foods on the Food Traceability List to maintain standardized records of critical tracking events (CTEs) and key data elements (KDEs). By specifying which data must be captured and how it must be maintained, the rule enhances the precision and speed of outbreak investigations. For example, the rule became effective on January 20, 2023, and FDA's analyses indicate it is expected to substantially shorten traceback times and improve the speed and precision of outbreak investigations; however, these benefits are presented in FDA's regulatory modeling and impact analyses rather than as observed post-implementation statistics (42). The emphasis on digital traceability also reflects a broader move toward modernized, technology-enabled food safety governance.

For PMDs, traceability is indispensable due to the complexity of their ingredient composition and processing. A single PMD may incorporate dozens of inputs sourced from multiple suppliers, processed at different sites, and distributed across extensive cold chains. Without robust traceability, identifying the source of contamination is extremely difficult, leading to delayed recalls and broader consumer exposure. International experience shows that mandatory, standardized traceability systems are essential for managing such risks effectively. For China, which currently relies on general legal obligations with limited enforcement, the establishment of a nationwide digital traceability system for PMDs would significantly strengthen consumer protection and regulatory capacity.

#### 3.3.5 Official controls and enforcement

International experience also highlights the importance of robust official controls and enforcement powers. In the EU, Regulation (EU) 2017/625 establishes a comprehensive framework for official controls across the food chain, covering inspections, audits, sampling, and testing. The regulation emphasizes risk-based prioritization, ensuring that resources are directed toward the most significant hazards. Crucially, it also provides authorities with strong sanctioning powers, including product recalls, suspension of operations, and financial penalties. These powers ensure that non-compliance carries consequences sufficient to deter violations (43).

In the US, FSMA similarly expanded the FDA's enforcement authority. Key powers include mandatory recall authority under Section 423 of the Federal Food, Drug, and Cosmetic Act and the ability to suspend facility registration under Section 415(b). These provisions empower the FDA to act decisively in cases where food poses a serious risk to public health, reducing reliance on voluntary recalls and negotiations with industry. By combining routine inspections with strong sanctioning mechanisms, the US creates an enforcement environment where compliance is not optional but a prerequisite for continued market participation.

For PMDs, where violations such as the use of substandard raw materials or falsification of records are recurrent risks, strong enforcement powers are critical. International practice demonstrates that enforcement must combine routine monitoring with the capacity to impose severe penalties when necessary (44). Without such deterrence, enterprises may calculate that non-compliance remains economically rational. For China, adopting stronger enforcement measures, including mandatory recalls and higher financial penalties, would align its regulatory framework with international standards and improve the credibility of its food safety governance.

#### 3.3.6 Rapid alert and digital modernization

Finally, international experience underscores the role of rapid alert systems and digital modernization in food safety governance (39). In the EU, the Rapid Alert System for Food and Feed (RASFF) facilitates real-time information exchange between Member States when food safety risks are detected. This system enables swift, coordinated responses, minimizing consumer exposure and harmonizing enforcement actions across borders. The transparency of RASFF also enhances public trust, as information on alerts and follow-up actions is made accessible to consumers and stakeholders.

In the US, the FDA has launched the New Era of Smarter Food Safety blueprint, which emphasizes the integration of digital technologies into food safety management. Priorities include tech-enabled traceability, data interoperability, and digital outbreak forensics. These initiatives recognize that the complexity of modern food supply chains requires solutions that go beyond paper-based records or localized databases. By leveraging digital tools, regulators can improve the speed and accuracy of risk detection, outbreak investigation, and recall execution (45).

For PMDs, which are characterized by multi-ingredient compositions and broad distribution networks, rapid alerts and digital traceability are especially important. Contamination in a single batch can quickly spread across provinces, making speed a decisive factor in risk management. International experience suggests that China would benefit from developing a dedicated rapid alert system for PMDs, integrated with a national digital traceability platform (46). Such a system would not only enhance regulatory efficiency but also strengthen consumer confidence by providing transparent and accessible information.

## 4 Discussion

### 4.1 From fragmentation to integration: a risk governance perspective

**Figure 1:**
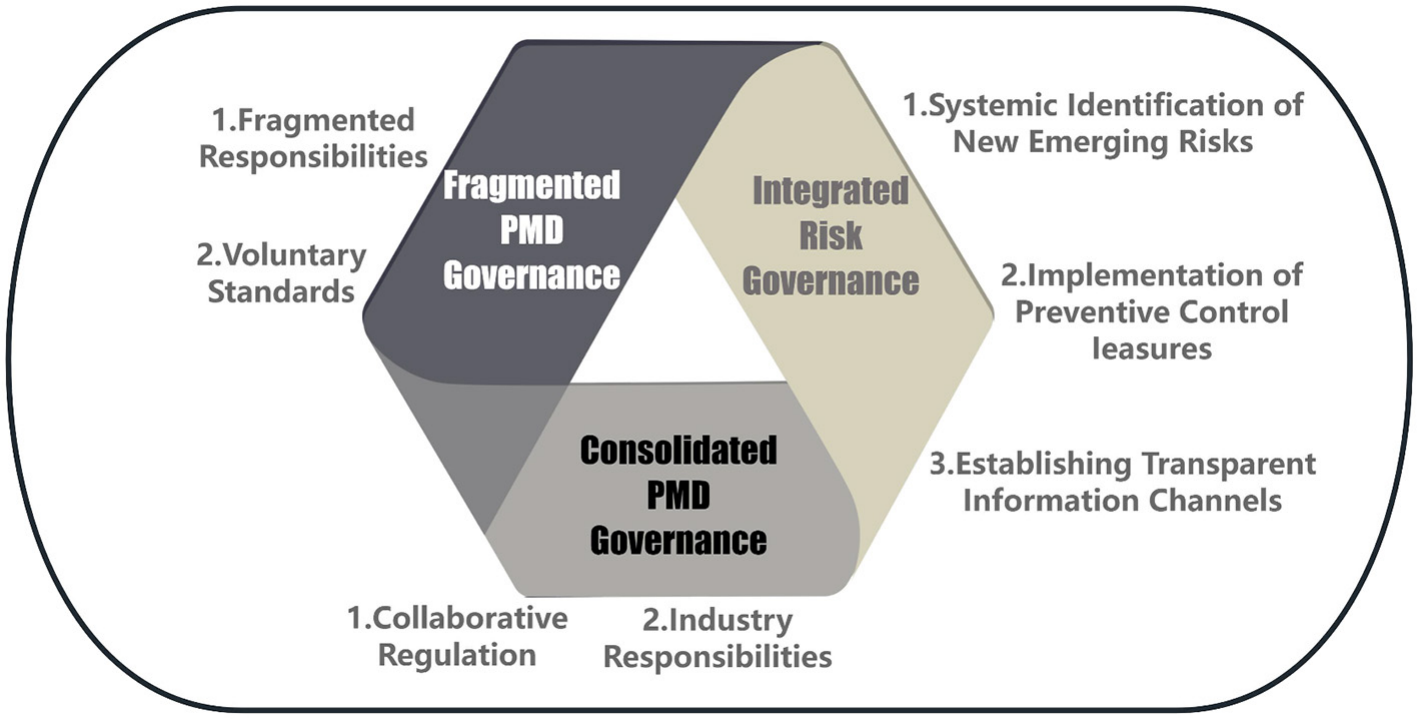
Implementation of integrated risk governance.

China's governance of PMDs reflects a fragmented institutional landscape in which responsibilities are dispersed across multiple levels of government, industry associations, and enterprises. As identified earlier, most standards remain voluntary, certification is limited, and traceability systems are inconsistently enforced. From the standpoint of risk governance theory, such fragmentation weakens the ability to anticipate, appraise, and manage systemic risks. According to the International Risk Governance Council (IRGC) framework, effective governance requires integration of five interrelated components: pre-assessment, risk appraisal, risk characterization, risk management, and risk communication. In the Chinese PMD context, these elements are not sequentially aligned; regulators prioritize ex-post inspections, enterprises often emphasize cost over preventive controls, and consumers are engaged primarily through crisis-driven media exposure rather than structured communication (see Figure 1).

This disjointed configuration has two critical implications. First, without integrated channels linking risk appraisal to management, emerging hazards in PMD production—such as microbial growth in cold chains or chemical residues in raw ingredients—may not be systematically identified or mitigated. Second, the absence of structured risk communication reduces transparency, thereby eroding consumer trust. By contrast, international practice demonstrates that embedding risk governance principles into law obligates enterprises to internalize hazard analysis and preventive controls, aligning corporate incentives with public health objectives. For China, transitioning from fragmented oversight to integrated risk governance requires not only codification of enterprise responsibilities but also institutionalized coordination mechanisms that connect state regulators, industry associations, and civil society actors. Establishing transparent information channels and participatory communication platforms would further ensure that risk management becomes anticipatory and preventive rather than reactive.

### 4.2 Polycentric governance and the challenge of coordination

**Figure 2:**
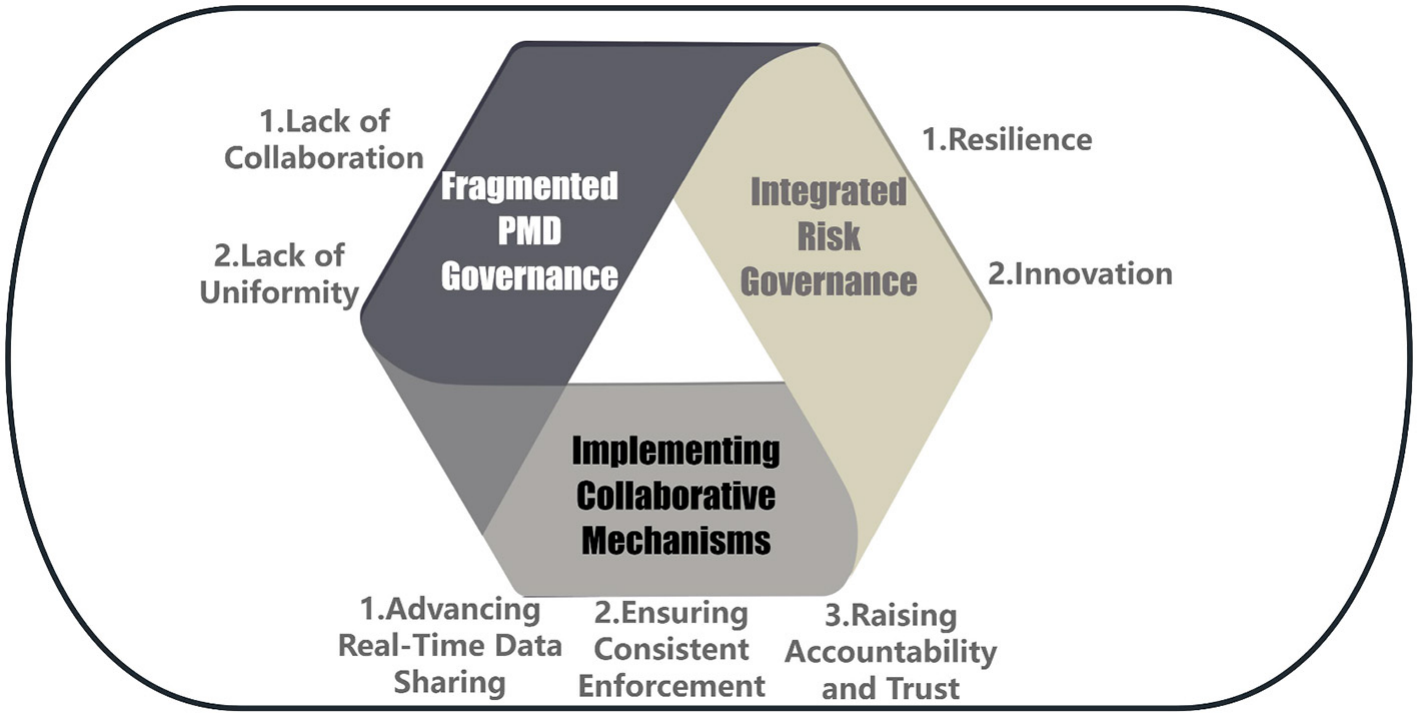
Improving China's PMD governance.

The governance of PMDs in China operates within a polycentric governance system, where authority and responsibility are distributed across multiple centers of decision-making. National ministries issue overarching laws, provincial governments pilot localized standards, industry associations set voluntary guidelines, enterprises engage in self-regulation, and consumers and the media influence reputational outcomes. As illustrated in Figure 2, these nodes form concentric circles of authority. In theory, polycentric governance can enhance resilience by enabling experimentation, innovation, and localized adaptation. Ostrom's scholarship highlights that polycentric arrangements are effective when nodes are interconnected through cooperative mechanisms that facilitate information exchange and mutual monitoring (47, 48).

In practice, however, China's PMD sector suffers from “polycentric fragmentation.” Pilot standards developed in provinces such as Shandong and Guangdong remain isolated, while industry associations promote group standards without binding authority. Overlapping responsibilities between food safety regulators at different levels create duplication in some domains and regulatory vacuums in others (45). The absence of effective coordination mechanisms means that innovations at the local or industry level rarely diffuse nationally, and accountability gaps persist. The result is a system that combines the costs of polycentricity—such as inconsistency and inefficiency—without reaping its potential benefits of adaptability and redundancy.

To transform this fragmented arrangement into a coherent polycentric system, institutional reforms must prioritize coordination. Mechanisms such as centralized digital traceability platforms, harmonized inspection protocols, and transparent reporting structures could link diverse governance nodes. By enabling real-time data sharing and joint enforcement, these tools would allow local governments, enterprises, and consumers to contribute to food safety governance while maintaining national coherence (see Figure 2). Such reforms would not eliminate polycentricity but instead recalibrate it, transforming diversity from a liability into a source of resilience and innovation in PMD governance.

### 4.3 Institutional isomorphism and pathways of reform

China's evolving governance of PMDs can also be interpreted through the lens of institutional isomorphism, a concept from neo-institutional theory that explains convergence toward similar organizational forms under coercive, mimetic, and normative pressures. Internationally, both the EU and US have institutionalized risk-based management, supply-chain verification, and mandatory traceability systems as binding legal requirements. For China, increasing integration into global markets and rising consumer expectations create strong coercive pressures to harmonize with these regimes, especially when trade requires adherence to HACCP or equivalent protocols (49).

Mimetic isomorphism is also visible, as Chinese policymakers emulate successful international practices to enhance domestic legitimacy. Pilot programs in Shandong on digital traceability and the adoption of enterprise-level HACCP certification illustrate attempts to replicate external models. At the same time, normative pressures are shaping industry behavior, as professional associations and international certification bodies promote standards such as ISO 22000. Together, these dynamics suggest that China's PMD governance will gradually converge with international frameworks.

However, convergence is not linear or uniform. Institutional path dependence and domestic political-economic considerations mean that China is more likely to engage in selective isomorphism. Rather than wholesale adoption of international models, reforms will be adapted to local contexts, balancing public health imperatives with industrial competitiveness. A plausible reform pathway involves integrating fragmented standards into a mandatory national baseline, embedding certification mechanisms akin to HACCP into regulatory law, and scaling up digital traceability nationwide. These reforms are not merely technical upgrades but institutional transformations that align China's governance with global norms while retaining distinctive features suited to its regulatory and industrial environment (47, 48). The challenge for policymakers lies in managing this convergence strategically, ensuring coherence and credibility while preserving flexibility for local adaptation (46).

### 4.4 Policy implications for China's PMD governance

The combination of diagnostic findings and international lessons yields several actionable policy implications. First, the establishment of a mandatory national standard for PMDs is imperative. For instance, in March 2024, the State Administration for Market Regulation, the Ministry of Education, the National Health Commission, and other relevant departments jointly issued the Notice on Strengthening Food Safety Supervision of Prepared Dishes and Promoting High-Quality Industrial Development. This document clarifies, for the first time at the national level, the definition and scope of prepared dishes. It also stipulates that the production, preparation, transportation, and storage of prepared dishes must comply with unified national standards, thereby enhancing consumers' sense of security and satisfaction with such products. Such a standard would replace the current patchwork of voluntary guidelines and local pilot schemes with a coherent framework applicable across all provinces. Second, certification mechanisms must evolve beyond production licensing to embed risk-based protocols into everyday operations. This would require SMEs, which dominate the PMD sector, to adopt structured safety management systems, potentially supported by subsidies or capacity-building programs. Third, the development of a national digital traceability system is urgently needed. While local pilots in provinces such as Shandong provide useful models, a centralized platform integrating QR codes, blockchain, and IoT technologies would create nationwide consistency and enable real-time oversight. Fourth, enforcement mechanisms must incorporate credible deterrence. Current fines and suspensions are insufficient to change enterprise behavior. International experience demonstrates the effectiveness of mandatory recalls, facility suspensions, and public disclosure of violations. China could adapt these tools to the PMD sector, ensuring that unsafe practices carry consequences that outweigh their economic benefits. Finally, policy reform should emphasize consumer empowerment. Transparency in labeling, traceability, and enforcement outcomes would allow consumers to make informed choices, creating market pressure for safer products (50). Together, these policy measures would not only address immediate governance deficiencies but also align the PMD sector with global best practices, thereby enhancing both domestic food security and international trade competitiveness.

### 4.5 Future research directions

While this study provides an integrated analysis of PMD governance, several areas warrant further investigation. First, future research could conduct comparative evaluations of local pilot programs, examining their effectiveness, scalability, and compatibility with national reforms. Empirical studies comparing provinces such as Shandong and Guangdong with regions lacking pilots would provide evidence for best practices and potential pitfalls. Second, quantitative risk modeling could identify the most critical control points in PMD production and distribution chains. By integrating microbiological risk assessment with supply-chain simulations, researchers could provide more precise guidance for regulatory interventions. Third, future studies should explore consumer trust and risk perception. PMDs are relatively new products in China, and consumer acceptance is closely tied to perceptions of safety and transparency. Cross-cultural research comparing Chinese consumers with those in the EU or US could reveal how regulatory frameworks influence trust. Fourth, interdisciplinary approaches that combine law, public health, data science, and behavioral economics would enrich understanding of how governance reforms translate into practical outcomes (51). Finally, longitudinal studies tracking the implementation of reforms could provide insights into how institutional isomorphism unfolds in practice, documenting both successes and unintended consequences. Addressing these research gaps would not only strengthen the evidence base for policymaking but also contribute to global scholarship on food safety governance in emerging markets.

## 5 Conclusions

This study has provided a systematic examination of the governance challenges surrounding pre-made dishes (PMDs) in China, drawing upon legal texts, regulatory standards, and empirical case analyses. The findings reveal a fragmented and uneven governance structure characterized by voluntary rather than mandatory standards, limited certification mechanisms, underdeveloped traceability systems, and inconsistencies in regulatory enforcement. These deficiencies collectively undermine food safety assurance, weaken consumer trust, and hinder the sustainable development of the PMD sector (50).

By situating China's experience within an international comparative perspective, the paper has highlighted how the European Union and the United States have institutionalized stronger frameworks through risk-based governance, enforceable supply-chain verification, mandatory traceability, and digital modernization (52). The comparison underscores the importance of shifting from reactive, fragmented oversight to a preventive and integrated governance model that embeds risk management into daily business practices. From a theoretical perspective, the study contributes to food safety governance literature by applying risk governance, polycentric governance, and institutional isomorphism frameworks to interpret China's PMD regulation. These perspectives help explain not only the persistence of regulatory fragmentation but also the drivers pushing toward convergence with international best practices. This theoretical elevation advances the debate beyond descriptive policy analysis toward a deeper understanding of institutional dynamics in emerging food sectors. The policy implications are equally clear. China should prioritize the establishment of mandatory national standards for PMDs, strengthen certification systems with binding risk-based requirements, and accelerate the rollout of a nationwide digital traceability platform (53). Moreover, regulatory enforcement should incorporate stronger deterrents, including mandatory recalls, transparent disclosure, and penalties proportionate to the risks of non-compliance. Such reforms would not only safeguard public health but also enhance consumer confidence and facilitate the integration of China's PMD industry into global food supply chains.

Finally, while this study provides a comprehensive diagnostic and comparative analysis, future research should deepen empirical investigations of local pilot programs, consumer risk perceptions, and the practical impacts of digital traceability innovations. Longitudinal studies tracking the implementation of governance reforms would be particularly valuable for understanding how institutional convergence unfolds in practice (54). By combining legal, technical, and social science perspectives, future scholarship can enrich both academic debate and policy design, ensuring that PMDs develop as a safe, trustworthy, and internationally competitive sector within China's evolving food system.
